# Coastal environmental changes in Ninh Thuan Province, South-Central Vietnam

**DOI:** 10.1371/journal.pone.0313382

**Published:** 2025-02-05

**Authors:** Bijeesh Kozhikkodan Veettil, Siham Acharki, Vikram Puri, Vanna Teck

**Affiliations:** 1 Laboratory of Ecology and Environmental Management, Science and Technology Advanced Institute, Van Lang University, Ho Chi Minh City, Vietnam; 2 Faculty of Applied Technology, School of Technology, Van Lang University, Ho Chi Minh City, Vietnam; 3 Center for Remote Sensing Applications (CRSA), Mohammed Virgin Islands Polytechnic University (UM6P), Benguerir, Morocco; 4 School of Computer Science, Duy Tan University, Da Nang, Vietnam; 5 Institute of Research and Development, Duy Tan University, Da Nang, Vietnam; 6 Faculty of Science and Technology, Svay Rieng University, Svay Rieng City, Cambodia; Center for Research and Technology Transfer, VIET NAM

## Abstract

Vietnam’s coastal regions are highly vulnerable to natural hazards and human-induced changes, posing significant challenges to their ecological and socio-economic systems. The country’s mangrove vegetation spans its entire coastline and has been depleted for decades in many regions. Notably, Vietnam’s proactive stance on climate change mitigation received significant recognition during the 26th Conference of the Parties (COP26) to the United Nations Framework Convention on Climate Change. This study investigated five critical coastal environmental features (shoreline dynamics, drought conditions, soil salinity trends, mangrove deforestation, and reforestation, as well as spatiotemporal variations in aquaculture and salt farming areas) using satellite data and geospatial analysis. Findings revealed a 58% decline in mangrove areas between 1989 and 2023, with a sharp decline between 1989 and 2001, followed by a gradual recovery. Furthermore, soil salinity along the Ninh Thuan coast indicated a continuous increase, except during the strong La Niña period in 2001. Additionally, aquaculture and salt marshes have expanded significantly, changing land use patterns. These findings highlight the urgent need for integrated coastal zone management to mitigate environmental degradation and enhance ecosystem resilience. Future studies should investigate the socio-economic implications of these changes and evaluate restoration strategies for sustainable coastal development.

## Introduction

Climate change and human activities have intensified coastal vulnerability globally, with regions in tropical and subtropical zones facing the most severe impacts. Vietnam is no exception; as one of the five countries most vulnerable to climate change, its coastal zones endure challenges such as shoreline erosion, rising sea levels, saltwater intrusion, and extreme weather events [1]. These issues are exacerbated by anthropogenic factors like deforestation, unregulated aquaculture, and inadequate environmental governance. Addressing these challenges requires immediate strategies such as the use of hard structures, such as seawalls and rock walls. For long-term coastal protection, ecosystem-based adaptation strategies using coastal vegetation bioshield or a combination of hard structures and coastal vegetation have proven effective [2]. However, several factors may influence the effectiveness of a coastal bioshield application, including geomorphological conditions of the coast, regional biodiversity, and the degree of vulnerability [2,3,4]. Among several coastal vegetation, mangroves are considered excellent coastal bioshields due to their adaptation to high salinity conditions and low soil oxygen content. Besides promoting sediment accumulation and protecting coastal shoreline against floods, and erosion caused by wave action [3], mangroves are effective bioshield against harmful UV radiation, hence protecting the fauna underneath the canopy [5]. The advantages of using mangroves as bioshield compared to hard structures are their cost-effectiveness, durability, and self-repairing ability after storm damage [6] as well as contributing to carbon sequestration.

The Vietnamese coastline hosts extensive mangrove forests, predominantly located in the Mekong Delta (with more than 70%) and the Red River Delta. However, these vital ecosystems have been significantly degraded due to the Vietnam War, extensive shrimp farming and illegal logging during the post-war period, and the historical deficiency in robust regulatory frameworks and governance mechanisms of mangrove ecosystem conservation and preservation [3,4]. Vietnam’s mangrove forests support fisheries and aquaculture, which are considered vital to the country’s economy, and they also support high species diversity and other ecosystem services [7,8]. However, the overexploitation of mangrove vegetation for exhaustive aquaculture ponds resulted in the loss of most Vietnamese mangroves, particularly on the southern coast [3,8]. Notwithstanding the substantial anthropogenic degradation observed in recent decades, Vietnamese mangrove ecosystems maintain their status as one of the globally significant biodiversity hotspots, characterized by exceptional primary productivity and remarkable ecological significance, protecting against storm events, providing aquatic ecosystems supporting both economically valuable and non-exploited ichthyofauna, and carbon storage (blue carbon) [3,9]. Similarly, Tran et al. [10] reported a 15.65% net increase in mangrove cover along the Southern Coast from 2016 to 2023 due to recovery programs and natural regeneration, despite significant industrial losses.

Restoration of mangrove vegetation is an ongoing task in various tropical nations around the world [11]. In Vietnam, several mangrove forest conservation efforts are carried out by various national and international organizations. However, the success (or failure) of these efforts is highly dependent on socioeconomic, political, and environmental factors [3,12], as well as climatological perturbations, eustatic sea level variations, meteorological disturbances, and oceanic circulation patterns. To compensate for the substantial depletion of Vietnam’s mangrove ecosystems, many restoration and rehabilitation programs have been, yielding varying levels of success [3]. Key reasons for the failure of many mangrove restoration initiatives in Vietnam are the lack of in-depth knowledge of underlying sediment and hydrological conditions, incorrect choice of species for planting, and conflicts among administrative bodies [3,13]. Moreover, financial compensation for mangrove conservation in Vietnam is limited. Nevertheless, a few successful mangrove restoration programs have been found in areas such as the Red River Delta, Ca Mau Peninsula, Can Gio mangrove biosphere reserve, and Kien Giang biosphere reserve [3]. Most of these initiatives were implemented on co-management schemes which improve the local population’s sense of ownership and responsibility [13,14].

Several research and review papers have extensively covered Vietnamese mangroves, particularly those from the two major river deltas [3,4,15]. For instance, Veettil et al. [3] discussed an in-depth analysis of the past and present conditions of Vietnamese mangrove forests about their geographical locations from the northeast to the south of the country’s coast. Most of the published papers have focused on areas such as the Mekong Delta, Can Gio Mangrove Biosphere Reserve, Ca Mau Peninsula, and the Red River Delta [3]. Many published research topics investigated the degradation, regeneration, and reforestation of mangrove vegetation, expansion of aquaculture ponds, and remote sensing of mangrove vegetation [2,3,16]. Remote sensing technologies, nonetheless, offer an economically viable methodological framework for cartographic documentation and surveillance of spatiotemporal dynamics within Vietnam’s mangrove ecosystems. Even though numerous remote sensing studies on these forests have been conducted in Vietnam, some sophisticated and updated technologies, such as the use of unmanned aerial vehicles (UAV) and high-resolution LiDAR data, are yet to be used to their full potential.

The south-central coast of Vietnam is reported to have faced drought conditions in recent years, particularly on the coast of Binh Thuan and Ninh Thuan provinces, due to climate changes [17,18]. Other contributing factors to the region’s desertification include inappropriate agricultural practices, deforestation, weak environmental management capacity, and lack of local awareness of the local population, as noted by Hai et al. [1]. For example, Tran et al. [19] highlighted that paddy fields in Binh Thuan declined by 51% in 2016 and the areas previously occupied by paddy fields have been transitioned to inactive status or occupied by the growth of drought-tolerant plant species, which is a sign of desertification. Recently, Nguyen et al. [20] observed signs of serious desertification in the southeastern regions of Ninh Thuan Province.

Remote Sensing constitutes a robust methodology for conducting temporal analyses of the aforementioned environmental perturbations in terrestrial surface characteristics. The primary objective of this study is to enhance the understanding of coastal environmental changes along Vietnam’s south-central coast. Specifically, it aims to: (a) identify the key drivers of shoreline dynamics; (b) evaluate the extent and impacts of drought on coastal ecosystems; (c) investigate changes in soil salinity and their implications for land use; (d) assess the progress and effectiveness of mangrove reforestation efforts; and (e) analyze the spatiotemporal expansion of aquaculture ponds and their environmental consequences. These insights are intended to inform sustainable coastal management and policy development, addressing critical challenges posed by climate change and human activities.

## Materials and methods

### Study area

**Fig 1:**
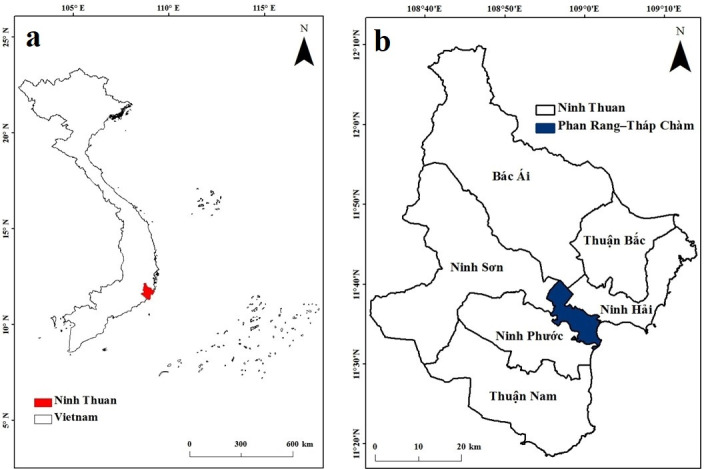
Geographical location of Ninh Thuan Province and the study area (coastal districts of Ninh Thuan).

Ninh Thuan (3,360 km^2^; 11°45′N 108°50′E) is a province along the south-central (or southeastern) coast of Vietnam (**Fig 1**). It comprises 6 districts and the provincial city of Phan Rang–Thap Cham and is considered as a region with low industrialization. The coastline of Ninh Thuan is 105 km in length and is rich in coastal environmental resources, including over 120 species of corals. The province has 116,172 hectares of protected forests which is about 59% of the total forest area in Ninh Thuan. It has been reported that the coastal region of Ninh Thuan is rich in metal minerals, such as wolfram, molybdenum, and tin, and non-metal minerals, such as quartz and porcelain. In addition, land acquisition is still going on for the installation of a nuclear power plant in Ninh Thuan, which will reduce the energy scarcity and dependence on fossil fuels for energy production in Vietnam [21].

Tropical monsoon climate prevails in Ninh Thuan and the province is considered a drought-prone province in Vietnam together with Binh Thuan Province in its southern border. The temporal precipitation distribution in Ninh Thuan exhibits a distinct bimodal pattern, characterized by an extended arid period spanning December through August, followed by a concentrated precipitation phase from September to November. The region demonstrates notable climatic extremes within the Vietnamese context, manifesting the highest thermal regime with mean annual temperatures ranging from 26 °C to 27 °C, concomitant with the nation’s minimum precipitation gradient. The spatial distribution of rainfall presents a marked altitudinal variation, ranging from 700–800 mm per annum in the urban administrative center to exceeding 1,100 mm in the elevated topographical zones [22] and humidity is around 75–77%. Despite the existence of a rainy season, Ninh Thuan has severe hydro-meteorological conditions, and the region is at risk of desertification and land degradation [23]. Essentially, Ninh Thuan is situated in the typhoon belt and has the lowest rainfall in Vietnam, coupled with the highest air temperatures.

**Fig 2:**
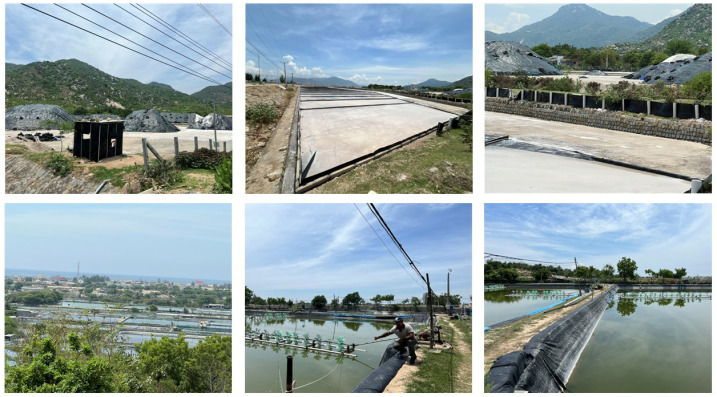
Major economic activities in coastal areas of Ninh Thuan (top: salt fields; bottom: aquaculture). Source: Dr. Bijeesh Kozhikkodan Veettil.

Most of the economic activities in Ninh Thuan are based on agriculture (rice and maize), forestry, and fishing as the province has one of the smallest industrial outputs (seafood, nuts) in the country. Several salt fields (**Fig 2**) can be found along the coastal districts of Ninh Thuan, particularly in Thuan Nam and Ninh Hai. Severe droughts that occurred in the past have caused significant damage to agriculture and other economic activities in the province [22]. According to the Ministry of Agriculture and Rural Development in Vietnam (MARD), water levels of rivers in many provinces, including Ninh Thuan, could be 20–50% lower in the coming year compared to the previous year [24]. In addition, there is a new port (Ca Na General Sea Port) under construction in Ninh Thuan.

Mangrove vegetation, although sparse, is found along the Ninh Thuan coast. In addition, seagrass beds and coral reefs were also found on the coast of Ninh Hai district [25,26]. Commonly found mangrove species in Ninh Thuan have been listed in **Table 1**. From 1999 and 2014, the area occupied by aquaculture ponds and mangrove vegetation increased by 78.18% and 0.06%, respectively [27]. Conversely, Quan et al. [28] estimated an increased land degradation in Nai Lagoon due to the expansion of aquaculture ponds and a dramatic decrease in mangrove vegetation between 1975 and 2014. Based on the world mangrove database [29], the extent of mangrove vegetation in Ninh Thuan is relatively low compared to the Mekong Delta in the south. Aquaculture expansion is intensive at the Nai Lagoon located in the northeast of Phan Rang in Ninh Hai district (**Fig 2–b**). Planting mangrove vegetation can be considered one of the best ways to improve the natural coastal environment, carbon sequestration, and shoreline protection [13] in Ninh Thuan.

**Table 1:** Commonly found mangrove/mangrove associate species found in Ninh Thuan Province, south-central Vietnam.

Family	Species	Vietnamese name(s)	Plant type	Salinity level
Aizoaceae	*Sesuvium portulacastrum* L.	Sam biển	Herb	Brackish/ sea water
Avicenniaceae	*Avicennia alba* Blume	Mắm trắng	Tree	Brackish
	*Avicennia marina* (Forsk.) Vierh.	Mắm biển	Tree	Brackish/ sea water
Rhizophoraceae	*Rhizophora apiculata* Bl.	Đước, đước đôi	Tree	Brackish
	*Rhizophora mucronata* Lam.	Đưng, đước bộp	Tree	Brackish/ sea water
Asteraceae	*Pluchea indica* (L.) Leres	Cúc tần, lức	Shrub	Freshwater/ brackish

Source: Veettil et al. [3]

### Data acquisition

Remote sensing methodologies utilizing multispectral satellite imagery present a cost-efficient approach for quantifying and analyzing coastal ecosystem dynamics, specifically pertaining to littoral erosional processes and modifications in mangrove vegetation distribution. This investigation employed multiple temporal sequences of cloud-free satellite imagery, including MODIS-derived Normalized Difference Vegetation Index (NDVI) data, successive generations of Landsat platforms (comprising Thematic Mapper, Enhanced Thematic Mapper Plus, and Operational Land Imager sensors), and Sentinel-2A multispectral observations to elucidate the spatiotemporal transformations along the Ninh Thuan coastal zone in south-central Vietnam. The MODIS NDVI data, with a spatial resolution of about 250 meters, provided insights into vegetation dynamics and drought conditions The spatial resolution characteristics of the utilized Earth observation datasets exhibit varying degrees of granularity: Landsat imagery provides a ground sampling distance of 30 meters in multispectral bands, with enhanced resolution of 15 meters in the panchromatic channel, while Seåntinel-2 data demonstrates heterogeneous spatial resolutions ranging from 10 to 60 meters, contingent upon the specific spectral bandwidth. These geospatial datasets are accessible via the United States Geological Survey’s Earth Explorer portal (https://earthexplorer.usgs.gov) without financial impediment. If required, atmospheric correction and conversion to surface reflectance were done for all multispectral data used. In conjunction with the aforementioned optical datasets, topographic information was incorporated through a digital elevation model (DEM) derived from the Shuttle Radar Topographic Mission (SRTM), exhibiting a 30-meter spatial resolution and accessible via the United States Geological Survey (USGS) repository. To authenticate the cartographic outputs generated from satellite-derived data, ground validation protocols were implemented through Global Positioning System (GPS) field surveys during two distinct seasonal periods: January 2020 (representative of arid conditions) and October 2021 (characteristic of pluvial conditions).

### Methodology

Environmental monitoring in Ninh Thuan Province, Vietnam, focuses on understanding shoreline changes, erosion-accretion dynamics, coastal vegetation changes, and soil salinity variations. Prior studies, such as those by Pham et al. [30], highlighted the region’s vulnerability to saltwater intrusion. Using Landsat and MODIS data, this research maps spatiotemporal variations in shoreline stability, aquaculture expansion, and vegetation changes, emphasizing spectral indices for analysis.

Spectral indices are integral to remote sensing analysis, offering insights into diverse environmental processes. The NDWI (Normalized Difference Water Index) is primarily employed to detect water bodies and delineate shorelines [31]. For assessing vegetation water content and monitoring drought conditions, researchers utilize NDMI (Normalized Difference Moisture Index), as shown in studies by Das et al. [32] and Sankey et al. [33]. Among all spectral indices, NDVI (Normalized Difference Vegetation Index) remains the most applied tool for vegetation assessment and mapping, according to Huang et al. [34]. Its inverse, NDSI (Normalized Difference Salinity Index), has proven valuable in monitoring soil salinity fluctuations, as evidenced in works by Khan et al. [35] and Tran et al. [36]. The CMRI (Combined Mangrove Recognition Index) specifically targets mangrove ecosystem identification in optical satellite imagery by analyzing the unique spectral signatures that distinguish mangrove vegetation from surrounding water bodies.

$$\text{NDWI =}\;\;\left[\text{Green – NIR}\right]\text{/ }\left[\text{Green +}\;\text{ NIR}\right]$$ (1)

$$\text{NDMI =}\;\;\left[\text{NIR -}\;\text{ SWIR}\right]\text{/ }\left[\text{NIR +}\;\text{ SWIR}\right]$$ (2)

$$\text{NDSI =}\;\;\left[\text{Red – NIR}\right]\text{/ }\left[\text{Red +}\;\text{ NIR}\right]$$ (3)

$$\text{NDVI =}\;\;\left[\text{NIR -}\;\text{ Red}\right]\text{/ }\left[\text{NIR +}\;\text{ Red}\right]$$ (4)

CMRI = NDVI – NDWI (5)

The Ninh Thuan shoreline’s spatiotemporal variations were analysed using NDWI, with a threshold segmentation for shoreline delineation. Even though good quality imagery without cloud cover is required, thresholding is the simplest image segmentation method with a rapid implementation [37]. Image differencing was applied to detect the areas undergoing erosion and accretion between two specific dates, using infrared channels (NIR and SWIR). Darker areas in the difference images indicate erosion, while brighter areas suggest accretion. Although this approach is effective for trend analysis, it is not quantitative (Gong 1993).

Ninh Thuan Province faces harsh hydro-meteorological conditions, exacerbating the risks of desertification and land degradation [23]. NDMI was used to monitor drought, complemented by spatiotemporal mapping of freshwater lakes using NDWI. The presence and surface area of lakes are critical drought indicators in tropical regions. Additionally, MODIS NDVI was applied to evaluate vegetation response to drought conditions, aligning with studies by Ji and Peters [38].

Soil salinity increase has been reported as a serious issue in southern Vietnam [39] due to saltwater intrusion [40]. NDSI, derived from Landsat data, was utilized to estimate soil salinity changes along the Ninh Thuan coast. While spectral reflectance-based methods provide qualitative salinity assessments, they offer a rapid and cost-effective approach compared to extensive field measurements [36,41].

In addition to the above-mentioned land cover features, the expansion of aquaculture, including salt ponds, was investigated using NDWI. It is worth noting that, depending on the investment cost, farmers have been shifting the crops (shrimp, lobster, snail, salt, etc.) in Ninh Thuan. Hence, only the overall changes in aquaculture ponds were mapped here instead of mapping distinct ponds, which is beyond the scope of remote sensing data used in this study.

Mangrove degradation and regeneration were analyzed using the CMRI and NDWI indices. These indices differentiate mangroves from other coastal vegetation based on spectral properties and water content [42]. Contemporary research initiatives, including the present investigation, have successfully employed NDWI for mangrove forest delineation and spectral differentiation from alternative vegetation classifications [4,42,43]. To enhance accuracy, the study incorporated Low Elevation Coastal Zone (LECZ) thresholds derived from DEM data, restricting mangrove analysis to elevations between 0–40 m [44,45]. This approach distinguishes mangroves from terrestrial vegetation occurring above the LECZ threshold.

**Fig 3:**
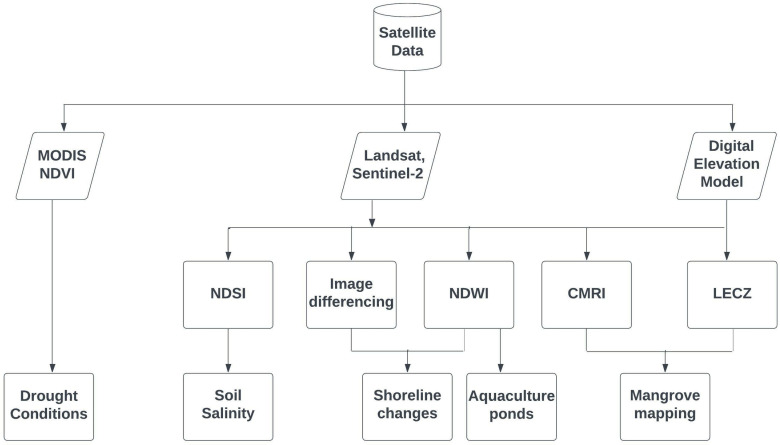
Flowchart of the methodology applied in the present study.

The overall methodology for estimating shoreline changes, drought characteristics, soil salinity changes, and mangrove degradation and regeneration in the study area is summarized in **Fig 3**.

## Results

**Fig 4:**
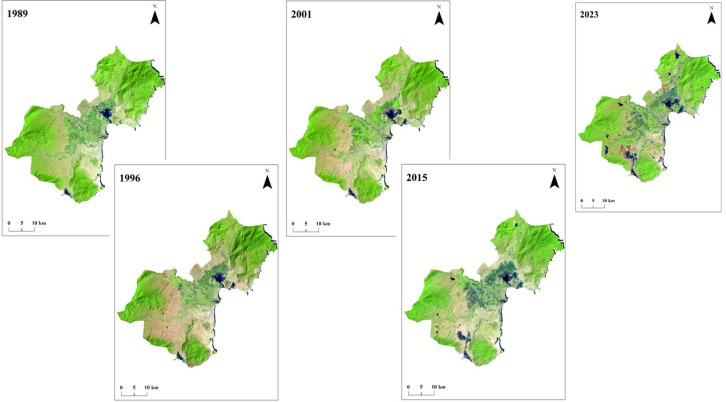
Satellite image series of the study site between 1989 and 2023.

The results of five environmental variables studied – shoreline changes, drought conditions, soil salinity changes, mangrove degeneration and reforestation, and aquaculture/salt ponds – showed a detailed picture of coastal environmental changes in Ninh Thuan over the last 3 decades. Visual inspection of the satellite image series of the Ninh Thuan coastal area (**Fig 4**) shows the expansion of aquaculture and salt farming areas between 1989 and 2023.

### Shoreline changes

**Fig 5:**
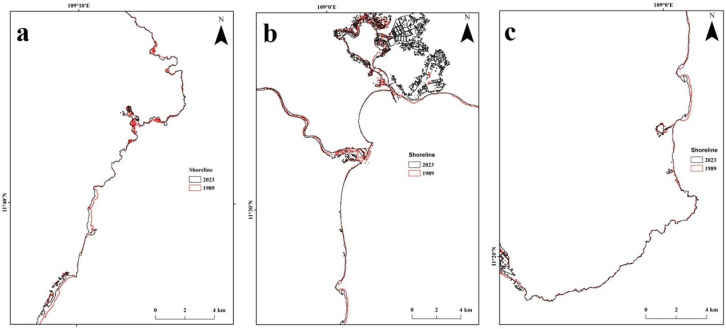
Shoreline changes along the coast of Ninh Thuan Province (a) Northern coast; (b) Central coast; (c) Southern coast.

The results indicated that shoreline changes between 1989 and 2023 were not uniform along the Ninh Thuan coast (**Fig 5**). The northern coast of Ninh Thuan (**Fig 5a**) did not show considerable erosion except in its southern region where the coastline has been eroded up to 90 m on average (maximum 120 m) from the previous shoreline. In addition, a considerable shift in shoreline occurred on the central coast of the province (**Fig 5b**), particularly in the north of the capital city (Phan Rang-Thap Cham). The southern coast of Ninh Thuan (**Fig 5c**) also showed no considerable changes in shoreline, except in its southern region.

### Drought conditions in Ninh Thuan

**Fig 6:**
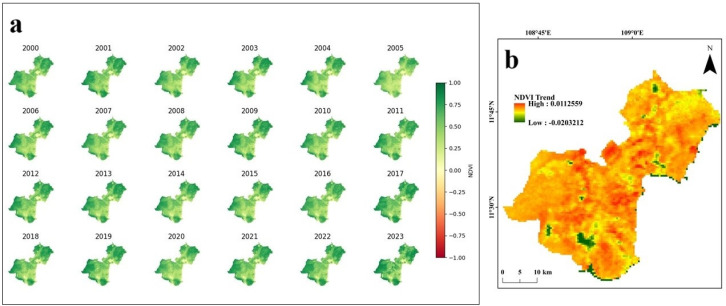
(a) NDVI time series between 2000 and 2023; (b) trends in NDVI between 2000 and 2023.

The analysis of MODIS NDVI trends from 2000 to 2023 in Ninh Thuan reveals significant spatial variability in the impact of droughts along the coast (**Fig 6**). The central and southern regions exhibit widespread areas of declining NDVI, indicating intense desertification and a considerable reduction in vegetation cover over the last twenty years. Conversely, the northern region presents a diverse pattern of NDVI trends, including both increasing and decreasing. This indicates that the region is responding to environmental conditions in different ways, with some zones recovering vegetation and others continuing to deteriorate. The coastal areas, especially near Vinh Phan Rang, show mixed NDVI trends, which are likely due to factors such as land use changes, saltwater intrusion, and water availability. **Fig 6** serves to highlight the crucial areas impacted by desertification and to indicate regions where vegetation recovery is possible. This provides essential insights for focused drought mitigation and land management in Ninh Thuan.

### Changes in mangrove vegetation

**Fig 7:**
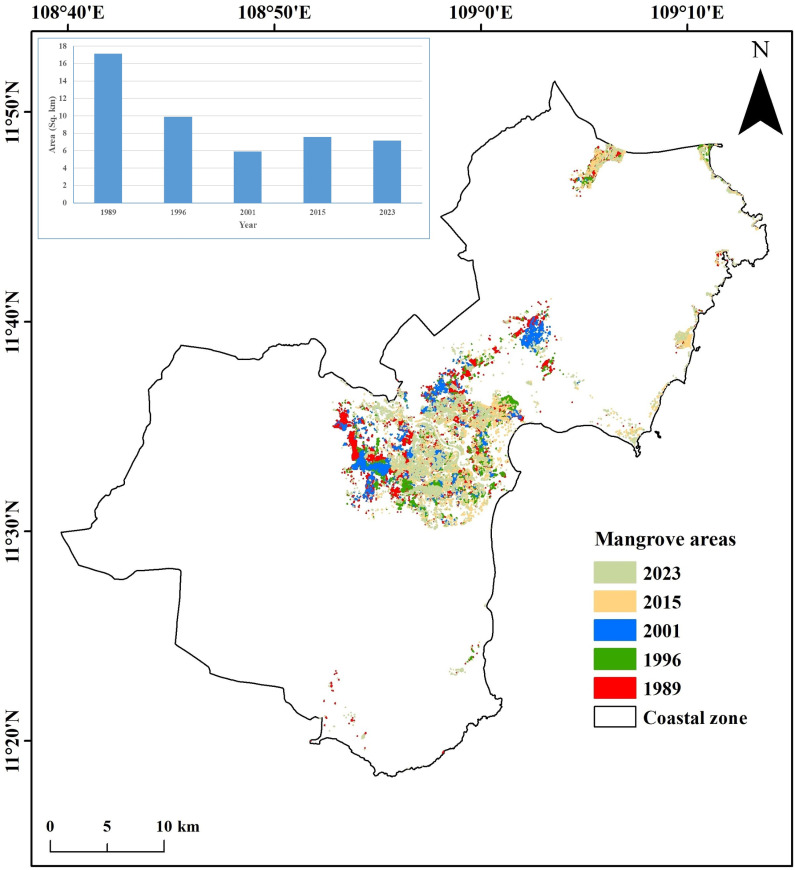
Spatiotemporal changes in mangrove vegetation (1989-2023) along the coast of Ninh Thuan province.

Our results indicated that even though mangrove vegetation was not widely distributed in Ninh Thuan province [3], the existing ones underwent degradation and loss in the area. There was an overall loss of 58.2% (from 17.13 km^2^ to 7.15 km^2^) of mangrove areas along the coastal zone of Ninh Thuan Province between 1989 and 2023 (**Fig 7**). Interestingly, there has been a rapid reduction in mangrove areas between 1989 and 2001, probably due to the expansion of aquaculture and salt farming areas. There was a slight increase in mangrove areas between 2001 and 2015, possibly due to mangrove reforestation as well as regeneration of mangroves. There has been a reduction in mangrove areas since 2015 to the present.

### Variations in soil salinity conditions

**Fig 8:**
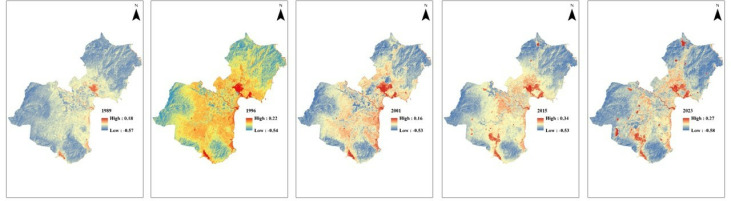
Soil salinity distribution between 1989 and 2023 using NDSI.

The analysis of soil salinity using NDSI, as shown in **Fig 8**, revealed variations in salinity levels across inland regions during 1989–2023, based on Landsat satellite data. However, it should be emphasized that soil salinity assessments derived from NDSI [35] are more reliable for qualitative evaluation rather than precise quantitative measurements. The observed differences in soil salinity in 1996, as compared to other years in **Fig 8**, can be attributed to climatic factors, particularly the influence of El Niño-Southern Oscillation (ENSO) events. Specifically, 1996 follows the significant El Niño event of 1997–1998, which is typically associated with higher temperatures, increased evaporation, and drier conditions, all of which can contribute to elevated soil salinity levels in the region. In contrast, 2001, which was marked by a prolonged La Niña period, likely brought cooler and wetter conditions that would reduce evaporation and result in lower salinity levels. However, research is required to understand the influence of El Niño-Southern Oscillation (ENSO) on soil salinity characteristics in this region, even though some correlation between soil salinity and El Niño events are observed in other areas of Vietnam, such as the Mekong Delta [46].

### Spatiotemporal changes in aquaculture and salt pan areas

Between 1989 and 2023, the total number and area of waterbodies (as observed in **Fig 4**), which consists of natural lakes, river channels, aquaculture ponds, and salt farming areas, have increased significantly in the coastal areas of Ninh Thuan Province (**Table 2**). Notably, the Ninh Hai and Thuan Nam districts have the highest number of shrimp ponds and salt farming areas. Since the 2000s, there has been an exponential increase in aquaculture and salt ponds in these areas. This observation agrees with the study conducted by Craeye [47], who observed the rapid increase in commercial shrimp farming areas in Ninh Thuan despite the lack of irrigation infrastructure.

**Table 2:** Spatiotemporal evolution of waterbodies in the coastal zone of Ninh Thuan Province.

Year	Number of waterbodies	Area of waterbodies (km^2^)
1989	315	15.07
1996	428	19.38
2001	762	20.69
2015	2070	39.61
2023	2883	49.2

## Discussion

The coastal and marine environments of Ninh Thuan are vital for the province’s economy, supporting agriculture, aquaculture, tourism, salt production, and diversified ecosystems. However, the coastal and marine environments of Ninh Thuan face significant challenges from climate change [48,49] and anthropogenic activities. Intensive shrimp farming and infrastructure development have exacerbated climate change impacts on Ninh Thuan and the central Vietnam coastlines, including increased frequency and intensity of droughts, saltwater intrusion, coastal erosion, and rising sea levels, which threaten livelihoods and ecosystems [50].

Notwithstanding the concurrent challenges posed by climatic perturbations and socioeconomic constraints, particularly during the SARS-CoV-2 pandemic period, Vietnam has maintained its resolute commitment to climate change mitigation strategies. The nation distinguishes itself as one of the inaugural twenty member states to submit an enhanced Nationally Determined Contribution (NDC) to the United Nations Framework Convention on Climate Change (UNFCCC) [51], demonstrating substantial progress in greenhouse gas emissions reduction initiatives through implemented policy mechanisms [52]. Vietnam’s initiatives in the fight against climate change have been appreciated by various international forums on climate change [51,52].

The province has implemented a forest land allocation program, assigning approximately 30 hectares per household for protection, with an annual compensation of 400,000 VND (US$17.4) per hectare. This initiative has facilitated the development of forest-linked livelihoods, including livestock rearing and fruit tree cultivation, thereby enhancing economic resilience among local communities. The local government is working to expand successful livelihood programs that connect communities with forest resources. However, forest management in Ninh Thuan continues to encounter various obstacles. These include limited funding, complicated regulatory frameworks, and vulnerability to climate conditions. The area’s characteristic hot and dry climate poses significant challenges for preventing forest fires, maintaining control measures, and establishing new forest areas. The challenging topography of the region also makes it difficult to effectively manage and protect forest resources. According to Đặng Kim Cương, who heads the provincial Department of Agriculture and Rural Development, plans have been developed to tackle these issues. These include enhancing public awareness of forest protection’s importance and legal frameworks, promoting sustainable forestry development, strengthening special-use, replacement, and protection forest programs, and improving inter-agency coordination for forest management.

The non-uniform changes in shoreline patterns show different environmental conditions along the Ninh Thuan coast. The vulnerability of the shoreline in northern areas of the Ninh Thuan coast shows the impacts of urbanization and expansion of aquaculture ponds and salt pans in this region and the clearing up of mangrove areas. This is evident along the coastal areas near the capital city of Phan Rang-Thamp Cham (**Fig 5a**). There has been a considerable increase in aquaculture farms and salt pans in this area, depleting mangrove vegetation that used to offer erosion protection and shoreline stabilization by accumulating sediments. Nevertheless, the protection offered by mangroves against shoreline protection is limited and cannot be considered for large-scale regional erosion [53].

It is crucial to note that the efficacy of these forestry initiatives is intrinsically linked to broader climatic patterns, particularly the El Niño-Southern Oscillation (ENSO). Research by Chung and Long [54] underscores the significant impact of ENSO conditions on drought and saltwater intrusion along Vietnam’s south-central coast. El Niño events, characterized by warmer sea surface temperatures in the central and eastern Pacific, typically correlate with reduced rainfall, exacerbating drought conditions and increasing saltwater intrusion risks. Conversely, La Niña phases, associated with cooler sea surface temperatures, often bring increased precipitation, potentially alleviating drought but elevating flood and soil erosion risks. These climatic variations highlight the critical need for spatiotemporal monitoring to predict and mitigate the impacts of ENSO-induced climate variability on Vietnam’s vulnerable coastal populations. The comprehensive approach adopted by Ninh Thuan province in forest conservation and expansion represents a significant step towards climate change adaptation. However, the success of these initiatives is contingent upon addressing ongoing challenges and considering the broader context of regional climate variability. Future research and policy development should focus on integrating advanced monitoring systems to enhance the resilience of Vietnam’s coastal ecosystems and communities in the face of climate change.

It is seen that about 65% of mangroves in Ninh Thuan, particularly in the Nai Lagoon, have been depleted between the 1989s and 2001 (from 17.13 km^2^ to 5.91 km^2^) due to various factors, such as the expansion of houses, infrastructures, and aquaculture ponds. The slight increase in mangrove areas between 2001 and 2015 is believed to have been influenced by reforestation activities and the regeneration of mangroves in abandoned aquaculture areas.

Aquaculture ponds, particularly brackish shrimp ponds, and salt farms have caused an increase in soil salinity conditions [55]. The contribution of the aquaculture industry to soil salinity changes is yet to be investigated along the south-central coast of Vietnam. Time-series remote sensing data allowed us to investigate such changes (**Fig 8**). The concurrent expansion of salt fields and aquaculture in Ninh Thuan reflects a dynamic economic landscape intertwined with significant environmental impacts. This necessitates careful management to sustainably balance economic growth with environmental conservation.

The present study showed that there was an exponential increase in the number and area of salt fields in the coastal zone of Ninh Thuan. Compared to many provinces in the southern region of Vietnam, the area occupied by salt fields in recent years is high in the study area. Since Ninh Thuan is the driest province with high salinity in the seawater, it appears to be one of the best places in Vietnam for salt production (Ninh Hai and Thuan Nam districts in particular). Recently, the farmers have used plastic sheets (**Fig 2** top) to produce salts, which is cleaner compared to traditional harvesting, which is slower and not cleaner. Moreover, salt farmers in Ninh Thuan have been linked with co-operatives in recent years to maintain steady prices and ensure markets. However, only a few studies, such as Ha [56], have estimated the presence of microplastics in salt samples from Vietnam, including from Ninh Thuan. It is visible from the results that the expansion of salt fields underwent at the cost of depletion of a few mangrove areas. However, it is widely believed that the environmental conditions in Ninh Thuan are not very suitable for extensive mangrove growth. In addition, many salt fields are located far from the nearshore areas, which can result in increased soil salinity in inland areas. Despite the economic benefits offered, several negative consequences are associated with the expansion of aquaculture ponds. These include soil acidity and salinity increase, pollution, eutrophication and nitrification of freshwater resources, invasive species accumulation, changes in natural hydrology, and habitat modification and loss of natural species [57].

There have been fewer studies in this region than in other areas such as the Mekong Delta [4]. These activities include shrimp farming, sandfish cultivation (sea cucumber – *Holothuria scabra*), and other salt-related enterprises. While aquaculture expansion offers economic opportunities, it also brings environmental challenges such as soil acidification, increased salinity, pollution, and habitat modification. The variability in net output from aquaculture ponds highlights the necessity of considering installation and maintenance costs when assessing the economic benefits of such activities.

## Limitations and future research directions

While this study offers valuable insights into the dynamics of land use changes, salinity variations, and the expansion of aquaculture and salt fields in Ninh Thuan, it has some limitations. The primary limitation is the reliance on remote sensing data, which, while effective for large-scale monitoring, may not capture finer local variations due to sensor resolution and cloud cover interference. Additionally, the study does not include detailed field-based soil salinity measurements, which could further validate the remote sensing results.

Future research should aim to combine remote sensing data with ground-truthing efforts to enhance the accuracy of salinity assessments. Further studies could investigate the long-term impacts of salt production and aquaculture on soil health, particularly the accumulation of microplastics and pollutants. Another promising area for future research is the development of predictive models to assess the future trajectory of coastal land use changes under varying climate scenarios, particularly the influence of El Niño and La Niña events. Expanding this research to include socioeconomic factors, such as the livelihoods of local communities and the economic sustainability of aquaculture, will help to create a more comprehensive understanding of the region’s resilience and adaptive capacity in the face of environmental and economic changes.

## Conclusions

The south-central coast of Vietnam, spanning from Da Nang to Binh Thuan provinces, is characterized by its unique climate and topography, and its narrow land area. In addition, the environmental conditions along the coastal areas of this region are not too favourable for mangrove vegetation. Nevertheless, mangrove patches were found in some provinces, such as in Ninh Thuan.

Shoreline changes were not uniform along the coast of Ninh Thuan province. While no considerable erosion occurred along the northern coast of Ninth Thuan, the southern coast eroded up to 90m to 120m in some areas. The shoreline near the capital city is also found to have eroded during the study period.

The analysis of MODIS NDVI trends from 2000 to 2023 in Ninh Thuan showed significant spatial variability in the impact of droughts along the south-central coast of Vietnam. The central and southern regions exhibit widespread areas of NDVI reduction, showing intense desertification and a considerable loss of vegetation between 2000 and 2023. In contrast, the northern region presents a diverse pattern of NDVI trends, including both increasing and decreasing values, indicating the region responding to environmental conditions in different ways. The coastal areas, especially near Vinh Phan Rang, show mixed NDVI trends, possibly due to factors such as land use changes, saltwater intrusion, and water availability.

Despite various afforestation initiatives, the total area covered by mangrove vegetation in Ninh Thuan Province of south-central Vietnam has decreased by 58.2% over the last 3 decades. It is hypothesised that regional climate and anthropogenic activities have influenced this observed reduction in sparsely existing mangrove areas in this region.

Since the early 2000s, there has been an exponential expansion of salt fields and aquaculture areas in Ninh Thuan Province. The number and area of waterbodies (mainly ponds) have been increased more than three times during the study period. If managed sustainably, the economy of the province could benefit from its salt production capability. Even though the unfavourable environmental conditions for mangrove vegetation, the presence of this vegetation may improve sustainable shrimp farming in the region. It is seen that soil salinity values showed an increasing trend along the coastal areas except for the year 2001, which was characterised by a prolonged La Niña period after the preceding El Niño period (1997–1998).

It is also worth noting that unmanned aerial vehicles (UAV) offer accurate and high-resolution map products in a cost-effective data acquisition procedure, making them suitable for small-scale coastline mapping, such as in Ninh Thuan province. The use of high-resolution UAV data is revolutionizing coastal environmental studies from remote sensing data.
