# Correction: Coastal environmental changes in Ninh Thuan Province, South-Central Vietnam

**DOI:** 10.1371/journal.pone.0330194

**Published:** 2025-08-12

**Authors:** The PLOS One Staff

There is an error in affiliation 3 for author Siham Acharki. The correct affiliation 3 is: Center for Remote Sensing Applications (CRSA), Mohammed VI Polytechnic University (UM6P), Benguerir, Morocco.

The publisher apologizes for the error.
